# Associations and interaction effects of socioeconomic, lifestyle, and genetic factors on intrinsic capacity

**DOI:** 10.1093/gerona/glag057

**Published:** 2026-02-23

**Authors:** Melkamu Bedimo Beyene, Renuka Visvanathan, Robel Alemu, Olga Theou, Beben Benyamin, Matteo Cesari, John Beard, Azmeraw T Amare

**Affiliations:** Adelaide Medical School, College of Health, Adelaide University, Adelaide, South Australia, Australia; Adelaide Geriatrics Training and Research with Aged Care Centre (GTRAC), The Basil Hetzel Institute, Adelaide University, Adelaide, South Australia, Australia; Adelaide Medical School, College of Health, Adelaide University, Adelaide, South Australia, Australia; Adelaide Geriatrics Training and Research with Aged Care Centre (GTRAC), The Basil Hetzel Institute, Adelaide University, Adelaide, South Australia, Australia; Aged and Extended Care Services, The Queen Elizabeth Hospital, Central Adelaide Local Health Network, Woodville, South Australia, Australia; Adelaide Medical School, College of Health, Adelaide University, Adelaide, South Australia, Australia; Anderson School of Management, University of California Los Angeles, Los Angeles, California, United States; Program in Medical and Population Genetics, Broad Institute of MIT and Harvard, Boston, Massachusetts, United States; Physiotherapy and Geriatric Medicine, Dalhousie University, Halifax, Nova Scotia, Canada; School of Public Health, College of Health, Adelaide University, Adelaide, South Australia, Australia; South Australian Health and Medical Research Institute, Adelaide, South Australia, Australia; Ageing and Health Unit, Department of Maternal, Newborn, Child & Adolescent Health and Ageing, World Health Organization, Geneva, Switzerland; Department of Clinical Sciences and Community Health, University of Milan, Milan, Italy; Robert N Butler, Columbia Aging Center, Columbia University Mailman School of Public Health, New York, New York, United States; Adelaide Medical School, College of Health, Adelaide University, Adelaide, South Australia, Australia; Adelaide Geriatrics Training and Research with Aged Care Centre (GTRAC), The Basil Hetzel Institute, Adelaide University, Adelaide, South Australia, Australia; (Biological Sciences Section)

**Keywords:** Intrinsic capacity, Aging, Polygenic score, CLSA, Lifestyle factors

## Abstract

Intrinsic capacity (IC), which reflects the combined physical and mental reserve of an individual, is a key indicator of healthy aging. While genetic and environmental factors influence IC, the interaction effects between them remain poorly understood. This study investigated the independent and interaction effects of polygenic scores for IC (PGS-IC), socioeconomic status, and lifestyle factors on IC. Baseline data from the Canadian Longitudinal Study on Aging (CLSA; *N* = 13 112) were analyzed. Associations with IC (outcome) and interaction effects of PGS-IC and socioeconomic factors or lifestyle measures — including composite physical activity and diet scores — were examined using linear regression models. All models were adjusted for age and sex and, additionally, for 5 genetic principal components in models involving PGS-IC. Higher IC was associated with higher education, income, physical activity, and healthier diet patterns. Lower IC was observed among previous and current smokers and those with short (<7 h) or long (>9 h) sleep durations. PGS-IC was positively associated with the IC score. Significant gene–environment interactions were identified between PGS-IC and Mediterranean diet (β = −.003, 95% CI, −0.006, −0.0002), education in younger adults (β = −.109, 95% CI, −0.211, −0.007), and sleep duration (younger adults: long sleep, β = .198, 95% CI, 0.023-0.373; older adults: short sleep, β = −.095, 95% CI, −0.153, −0.036). This study provides preliminary evidence of gene–environment interactions influencing IC, with implications for future research to determine how genetic and modifiable factors can inform strategies for maintaining IC and promoting healthy aging.

## Introduction

With population aging observed globally at an unprecedented rate, maintaining health and functionality in older age is a public health priority.^1^ Traditional approaches to aging have predominantly focused on disease management and morbidity reduction. However, there is a growing consensus that a broader preventative approach across the lifespan is needed — one that maximizes functional ability in older age, enabling older people to be functionally able, continuing to do what they value as they age.^1–3^ The World Health Organization (WHO), within its healthy aging framework, outlined that functional ability is influenced by intrinsic capacity (IC), the environment (eg, socio-economic and lifestyle factors), and the interaction of these 2 factors.^1^^,^^4^ IC is operationalized through 5 key domains — locomotion, vitality, cognition, psychological well-being, and sensory function.^1^^,^^4–6^

There is growing evidence that IC itself is influenced by a dynamic interplay between genetic predisposition and external factors, including socioeconomic, lifestyle, and behavioral influences.^1^^,^^7^^,^^8^ Our scoping review^9^ identified a wide range of socioeconomic and lifestyle factors associated with IC, such as educational, social, and economic status, engagement in social activities, and other lifestyle factors such as diet, exercise, physical activity, smoking, and sleep. Building upon this, our subsequent research program using the Canadian Longitudinal Study on Aging (CLSA) and UK Biobank data revealed that a quarter of the interindividual variability in IC is attributable to genetic variation and that the polygenic score for IC (PGS-IC) in CLSA was associated with the IC scores.^8^ Understanding the consistency of this polygenic association across age groups, and how genetic risk interacts with socioeconomic and lifestyle factors to influence IC is an important next step in our research program. Such insights could help identify potential targets for intervention, particularly modifiable factors that could preserve IC and support healthy aging.

Therefore, the primary aim of this study was to examine the cross-sectional association of IC with socio-economic/lifestyle factors, and the PGS-IC when stratified by age, and explore the interaction effects between PGS-IC and these factors on IC.

## Methods

### Study cohort: the CLSA

This study leveraged data from the CLSA, the largest nationally representative cohort of aging adults in Canada. The CLSA includes over 50 000 participants aged 45-85 years at baseline, recruited from various regions across Canada except for the 3 northern territories and remote areas of Canada that are difficult to access, encompassing both urban and rural settings.^10^^,^^11^

#### Inclusion and exclusion criteria

Participants were included in the CLSA study if they could complete interviews in English or French and had sufficient physical and cognitive capacity to take part independently, including being able to hear and respond to the study questions.^10^^,^^11^ People residing on Federal First Nations reserves or other First Nations settlements, full-time members of the Canadian Armed Forces, and individuals living in institutional settings such as long-term care facilities were excluded.

The cohort was divided into 2 subgroups: the tracking cohort and the comprehensive cohort. For tracking cohort, data were collected through telephone interviews across the 10 provinces of Canada, whereas the comprehensive cohort participants underwent in-person physical assessments and provided blood and urine samples at 11 data collection sites.

#### Study sample

For this analysis, we focused on the baseline socioeconomic and lifestyle variables of the comprehensive cohort, which comprises more than 30 000 community-dwelling adults who underwent in-depth physical, clinical, and biological assessments. Of these, genetic data were available for 26 622 participants.^12^ After quality control procedures, a final sample of 13 112 individuals with complete data on IC variables and genetic information was retained for analysis in this study.

#### Outcome variable (IC score)

We have previously described the development and validation of the IC score for the CLSA.^8^ Briefly, the IC scores were constructed using 14 baseline variables and by applying a factor analysis approach, specifically the bifactor approach, as described in the Supplementary Methods. The resulting score demonstrated strong construct and predictive validity, showing expected associations with key health outcomes (eg, age, sex, and mortality) and effect directions consistent with prior evidence.^8^^,^^9^ Prior to the data analysis, the final IC score generated for the general IC factor was standardized (converted to a Z-score) to approximate a normal distribution with a mean of zero and a *SD* of 1, facilitating comparability and subsequent statistical modeling.

### Socioeconomic and lifestyle factors

The socioeconomic variables included educational attainment (assessed by graduation from high school, highest degree achieved, and highest elementary or high school completed), employment history (working status — yes/no at the time of interview), income (assessed by total annual personal income in Canadian dollars — measured in 5 categories), and social engagement (playing games and musical instruments/singing), and the lifestyle variables were diet, physical activity, smoking, and sleep health. The complete list of variables included in the analysis can be found in Tables 1 and 2. These factors were selected based on our previous literature review, which outlined the list of socioeconomic and lifestyle factors strongly associated with a wide range of health and functional ability domains.^9^ To evaluate the cumulative and broader effects of individual lifestyle factors, we computed composite measures of diet (Prospective Urban Rural Epidemiological [PURE] healthy diet score and Mediterranean diet scores) and physical activity (Physical Activity Scale for the Elderly [PASE] scale) and assessed their potential associations with IC.^13–16^

#### Diet scores

Nutritional assessment in the CLSA was conducted using the Short Diet Questionnaire (SDQ), a validated tool designed to provide an overview of dietary habits (quantity and frequency of consumption) of the study participants.^17^ Using these data, we constructed 2 widely used and validated composite dietary indices: the PURE healthy diet score^14^ and the Mediterranean diet score.^18^

The PURE healthy diet score was calculated based on the frequency of consumption (converted to the number of times of intake per day) of 7 healthy food groups: fruits, vegetables, legumes, nuts, fish, dairy, and meats (including red meat, pork, and poultry). Each food group was scored on a quintile-based scale ranging from 0 to 4, with higher scores indicating healthier intake levels. The final score, obtained by summing the scores across all 7 groups, ranged from 0 to 28, representing the spectrum from poorest to best diet quality.^14^^,^^19^ Detailed components of the PURE healthy diet are provided in the Supplementary Methods. Similarly, the Mediterranean diet score was calculated using the frequency of consumption (converted to the number of times of intake per day) for 10 food/drink groups based on CLSA SDQ data.^18^^,^^20^ Beneficial components (whole grains, fruits, vegetables, legumes, nuts, potatoes, and fish) were positively scored, while detrimental components (meat and meat products, poultry, full-fat dairy, and alcohol) were inversely scored. Each group contributed 0-5 points, yielding a total score from 0 to 50, with higher scores indicating greater adherence to the Mediterranean diet.^18^^,^^20^ Full scoring details are provided in the Supplementary Methods.

The *PASE* is a validated tool for evaluating the frequency and intensity of physical activities in older adults.^21^ The total PASE score (0-400+) was derived as the sum of weighted scores from 12 self-reported items covering leisure, household, and occupational activities over the past 7 days, based on the original PASE scoring manual.^21–23^ Full details of scoring procedures and variable derivations are provided in the Supplementary Methods.

#### Sleep

Sleep duration was assessed based on participants’ self-reported average hours of sleep in a 24-h period. Later, we categorized them into 3 groups: short sleep (<7 h), optimal/recommended sleep (7-9 h), and long sleep (>9 h), in line with established sleep health guidelines.^24^ In addition, we have also tested trouble falling asleep, difficulty staying awake during the day, as well as satisfaction with sleep patterns.

### Polygenic score for IC

Polygenic score for intrinsic capacity (PGS-IC) was developed in our previous study^8^ for CLSA participants (target cohort) using the Polygenic Risk Score-Continuous Shrinkage method,^25^ which has been shown to yield higher predictive performance compared to other approaches.^26–28^ This method applies a Bayesian regression framework with continuous shrinkage priors to estimate the posterior effect sizes of single-nucleotide polymorphisms (SNPs). The scores were computed using GWAS summary statistics from the UK Biobank (discovery dataset)^8^ and linkage disequilibrium (LD) reference data from the European panel of the 1000 Genomes Project^29^ to match the predominantly European ancestry of the CLSA sample (97% for our sample) and minimize LD mismatch. A total of 652 994 SNPs were included in the PGS calculation.

### Statistical analysis

Descriptive statistics were used to summarize the study variables. Categorical variables were summarized using counts (*n*) and proportions (%), while continuous variables were summarized using means and *SD*s.

This study employed 3 independent linear regression models to examine the association of IC with: (1) socioeconomic or lifestyle factors, adjusting for age and sex (Model 1); (2) PGS-IC adjusting for age, sex, and the first 5 genetic principal components (PCs) (Model 2) followed by stratification by age groups 45-64 and 65+; and (3) the interaction of PGS-IC and socioeconomic/lifestyle factors, adjusting for the main effect of each socioeconomic/lifestyle factor, the PGS-IC, age, sex, and the first 5 PCs (Model 3). The interaction effect analyses were undertaken for (1) the entire sample and (2) the sample stratified by 2 age groups: 45-64 and 65+, because there was a differing pattern in the association of PGS-IC with the IC score in our preliminary analysis. The age categorization for model 2 (45-64 and 65+ years) was based on previous evidence that IC follows a 3-phase life-course trajectory: (1) an increasing pattern from childhood to early adulthood, (2) a high and relatively stable plateau during mid-life, and (3) a subsequent decline beginning in older age, with the inflection point for this decline usually occurring around 65 years.^1^^,^^30^^,^^31^ As the CLSA sample comprises adults aged 45-85 years, the age categories correspond to the latter 2 phases of the IC life-course trajectory. Accordingly, for the age-stratified analysis, participants were grouped into 2 age categories: 45-65 years and ≥65 years.

For models with significant interaction effects (either in the whole sample or age-stratified analyses), separate linear regression analyses were done to compare the effect of the corresponding socioeconomic or lifestyle factor in individuals with low (decile [D1]), middle (D2-D9), or high (D10) PGS-IC.

Decile-based regression analyses were conducted for the 3 lifestyle factors — Mediterranean diet score, PURE healthy diet score, and PASE. Each composite score variable was divided into 10 deciles based on its distribution in the sample. The lowest diet or physical activity score was D1, with ascending order to the highest score at D10. Multivariable linear regression models were run to test associations of these diet and physical activity scores with IC and estimate β-coefficients with 95% CIs for individuals with high composite diet or physical activity scores (D2-D10) compared to those with poor diet or physical activity habits (lowest decile: D1, as the reference group). All regression models were adjusted for age and sex and, for PGS-related models, additionally for the top 5 genetic PCs. The analyses were conducted using *lm* function from the “*stats*” package in R (version 4.3.1).

The associations between each lifestyle and socioeconomic factors and IC were tested independently using separate models, with adjustment for key confounders. The choice of confounding variables was based on existing literature indicating that many lifestyle and socioeconomic factors are strongly interrelated; simultaneous adjustment for all such factors may therefore introduce multicollinearity and lead to overadjustment.^14^^,^^32–37^

Multiple testing corrections were applied using the Benjamini–Hochberg False Discovery Rate procedure. Associations were considered statistically significant at an FDR-adjusted *p*-value <.05.

**Model 1**: IC = β_0_ + β_SLFi_ + β_Age_ + β_Sex_ + εi

**Model 2**: IC = β_0_ + β_PGS-IC_ + β_Age_ + β_Sex_ + $\gamma^{\prime }$PC_1-5_ + εi

**Model 3**: IC = β_0_ + β_PGS-IC_ + β_SLFi _+ β_interaction_  _(PGS-IC x SLFi)_ + β_Age_ + β_Sex_ + $\gamma^{\prime }$PC_1-5_ + εi

where IC = intrinsic capacity, PGS-IC = polygenic score for IC, β = beta coefficient, SLF = Socioeconomic or lifestyle factors, $\gamma^{\prime }$PC_1-5_ is a vector of the first 5 genetic PCs, and εi are error terms.

## Results

The mean (*SD*) age of the sample was 61 (9.6) years, and 50.8% of participants were female. The cohort had predominantly European (97.4%) genetic ancestry, with smaller representations of Asian (1.4%), African (0.6%), Hispanic (0.4%), and other genetic backgrounds (0.2%). The description of sociodemographic characteristics and lifestyle factors are detailed in Tables S1 and S2.

### The association of IC with socioeconomic and lifestyle factors

#### Socioeconomic factors

Among the socioeconomic factors, current employment status, educational attainment, personal income, and participation in leisure activities, such as playing games and music, were significantly associated with IC. For example, participants who reported being employed (working) at baseline had significantly higher IC scores than those who were not working (β = .188, 95% CI, 0.121-0.255). Educational attainment, assessed by the highest school completed, graduation from high school, as well as the highest degree achieved, all showed consistent positive associations with IC (Table 1), with higher levels of education being associated with higher IC scores. For example, individuals who had graduated from high school have higher IC scores than those who had not (β = .255, 95% CI, 0.180-0.329). Total personal income also showed a consistent positive association with IC, with all higher income categories demonstrating significantly higher IC scores compared with those in the lowest income group. Similarly, engagement in leisure activities, such as playing games and musical instruments, was positively associated with IC, with greater frequency of participation relating to higher IC scores (Table 1).

**Table 1:** The association of IC with socioeconomic factors (SEFs) and the interaction of SEFs and PGS-IC.

Variable/category	Categories	Main effect: β (95% CI)	Interaction effect: β (95% CI)
**Education**			
Graduated from high school	Graduated vs. Not	.255* (.180, .329)	−.065 (−.139, .009)
Highest degree achieved	Trade certificate/diploma vs. No Post.sec	−.146* (−.211, −.081)	−.029 (−.095, .037)
	Non-uni. certificate/diploma vs. No Post.sec	−.017 (−.076, .042)	−.019 (−.077, .039)
	University certificate vs. No Post.sec	.062 (−.020, .143)	−.081 (−.160, −.003)
	Bachelor’s degree vs. No Post.sec	.225* (.169, .282)	−.033 (−.089, .022)
	Uni. degree/cert. above BA vs. No Post.sec	.340* (.283, .397)	−.046 (−.102, .010)
Highest elementary or high school completed	Grade 9-10 vs. Grade 8 or lower	.138 (−.005, .281)	−.074 (−.214, .066)
	Grade 11-13 vs. Grade 8 or lower	.680* (.557, .803)	−.004 (−.125, .117)
**Employment and income**			
Currently working	Working vs. Not working	.188* (.121, .255)	.018 (−.045, .081)
Total personal income	$20 000-49 999 vs. Less than $20 000	.157* (.111, .202)	.022 (−.023, .066)
	$50 000-99 999 vs. Less than $20 000	.253* (.207, .299)	.014 (−.030, .058)
	$100 000-149 999 vs. Less than $20 000	.358* (.298, .418)	.014 (−.044, .071)
	$150 000+ vs. Less than $20 000	.392* (.322, .461)	.042 (−.024, .108)
**Engagement — social/leisure activity**			
Play games, cards, etc	Several times a year vs. Once a year or less	.095* (.045, .145)	−.018 (−.068, .032)
	Several times a month vs. Once a year or less	.068* (.020, .115)	−.023 (−.069, .024)
	Several times a week vs. Once a year or less	.097* (.054, .141)	−.007 (−.051, .036)
	Every day vs. Once a year or less	.122* (.083, .161)	−.023 (−.061, .015)
Play a musical instrument or sing	Several times a year vs. Once a year or less	.181* (.114, .249)	−.010 (−.074, .055)
	Several times a month vs. Once a year or less	.188* (.131, .244)	−.021 (−.076, .034)
	Several times a week vs. Once a year or less	.246* (.194, .298)	−.020 (−.072, .032)
	Every day vs. Once a year or less	.283* (.211, .355)	.006 (−.065, .076)

Values are effect estimates (β) with 95% CI for associations between SEFs and IC, and the interaction effect of SEFs and PGS on IC.

Abbreviations: PGS-IC, polygenic score for IC, vs., versus, FDR, false discovery rate; IC, intrinsic capacity; PGS, polygenic scores; β, beta coefficient.

* Statistically significant associations (*p*_FDR-adjusted_ < .05).

#### Diet types and composite diet scores

**Figure 1:**
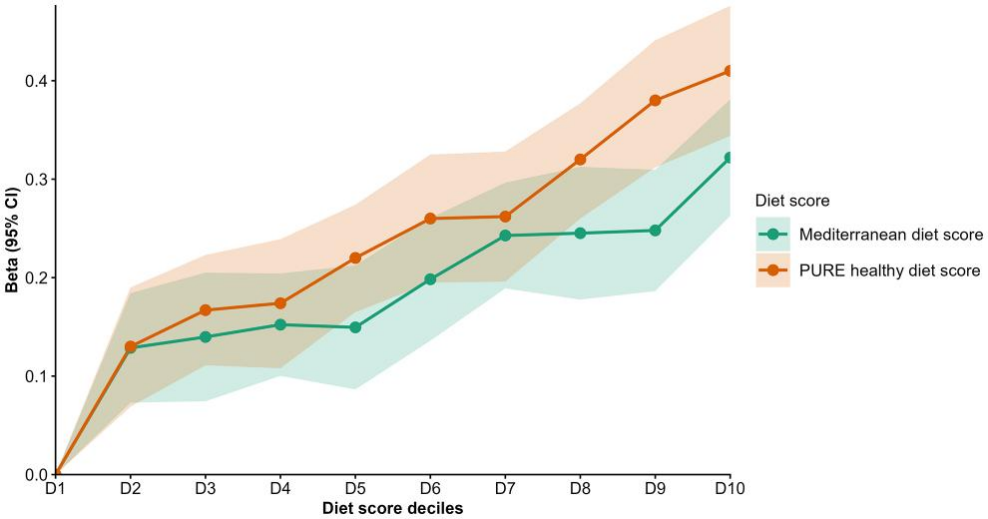
Association between IC and healthy diet scores (across deciles) in the CLSA cohort. D = decile (D1 [lowest] to D10 [highest]), beta = regression coefficient for individuals with high diet scores (i.e., Deciles D2-D10) compared with those at the lowest diet decile (D1). CLSA, Canadian Longitudinal Study on Aging; IC, intrinsic capacity; PURE, Prospective Urban Rural Epidemiological study.

Dietary intake was significantly associated with IC, with detailed results for individual food types provided in Table S3. Moreover, the 2 composite healthy diet scores assessed showed strong associations with IC. A higher PURE healthy diet score (β = .024; 95% CI, 0.021-0.027) and greater adherence to the Mediterranean diet (β = .018; 95% CI, 0.016-0.022) were both associated with higher IC. Decile-based stratified analyses support these findings with a clear dose–response relationship where IC scores increased consistently across increasing deciles of both diet scores (Figure 1). Detailed results for the association of lifestyle factors with IC are provided in Table 2.

**Table 2:** Association of IC with lifestyle factors and the interaction of lifestyle factors and PGS-IC.

Variable/category	Categories	Main effect: β (95% CI)	Interaction effect: β (95% CI)
**Dietary quality**			
PURE healthy diet score	Score (0-28)	.024* (.021, .027)	−.002 (−.004, .001)
Mediterranean diet score	Score (0-50)	.018* (.016, .021)	−.003* (−**.**006, −.0002)
**Physical activity**			
PASE score	Score (0-400+)	.0006* (.0004, .0008)	−.0001 (−.0003, .0001)
**Smoking**			
Smoking status	Previous smoker vs. Never smoker	−.093* (−.121, −.064)	−.011 (−.039, .017)
	Current smoker vs. Never smoker	−.407* (−.459, −.355)	.033 (−.018, .084)
Current frequency of cigarettes smoked	Occasionally vs. Never	−.123* (−.230, −.017)	−.003 (−.115, .110)
	Daily vs. Never	−.403* (−.463, −.343)	.046 (−.012, .104)
Current No. of cigarettes smoked/day	6-10 cigarettes vs. 1-5 cigarettes	−.104 (−.297, .090)	−.025 (−.206, .155)
	11-15 cigarettes vs. 1-5 cigarettes	−.098 (−.294, .099)	.046 (−.130, .222)
	16-20 cigarettes vs. 1-5 cigarettes	−.198 (−.410, .013)	.163 (−.036, .363)
	21-25 cigarettes vs. 1-5 cigarettes	−.188 (−.425, .050)	.161 (−.061, .384)
	26+ cigarettes vs. 1-5 cigarettes	−.709* (−1.036, −.383)	.103 (−.207, .412)
Frequency of usual passive smoking exposure at home	Once a week or less vs. Never	−.283* (−.373, −.193)	.012 (−.069, .093)
	Every day or almost every day vs. Never	−.422* (−.512, −.332)	.101*(.003, .199)
**Sleep**			
Sleep duration (h/day)	Sleep hours per day	.043 (.031, .054)	.006 (−.006, .018)
Sleep duration (categories)	Short (<7 h) vs. Optimal (7-9 h)	−.133* (−.161, −.105)	−.021 (−.049, .007)
	Long (>9 h) vs. Optimal (7-9 h)	−.258* (−.392, −.124)	.026 (−.110, .161)
Frequency of difficulty staying awake during the day	Less than once/week vs. Never	.024 (−.011, .058)	.011 (−.023, .046)
	Once or twice/week vs. Never	−.017 (−.064, .031)	−.004 (−.051, .043)
	3-5 times/week vs. Never	−.073* (−.139, −.007)	.040 (−.026, .107)
	6-7 times/week vs. Never	−.241* (−.323, −.159)	−.095* (−.174, −.016)
Satisfaction with current sleep pattern	Dissatisfied vs. Very dissatisfied	.075 (−.003, .153)	−.025 (−.103, .053)
	Neutral vs. Very dissatisfied	.074 (−.005, .154)	−.022 (−.101, .058)
	Satisfied vs. Very dissatisfied	.075* (.0001, .150)	−.007 (−.083, .068)
	Very satisfied vs. Very dissatisfied	.120* (.042, .198)	−.005 (−.083, .073)

Values in the table are effect estimates; betas (β) with 95% CI for associations between lifestyle factors and IC, and the interaction effect of lifestyle factors and PGS on IC.

Abbreviations: PGS-IC, polygenic score for IC, vs., versus, FDR, false discovery rate; IC, intrinsic capacity; PASE, Physical Activity Scale for the Elderly; PGS, polygenic scores; PURE, Prospective Urban Rural Epidemiological study; β, beta coefficient.

* Statistically significant associations (*p*_FDR-adjusted_ < .05).

#### Cigarette smoking

In this analysis, all the cigarette-smoking-related variables (smoking status, number of cigarettes smoked, and frequency of smoking) showed consistent negative associations with IC. Current smokers (β = −.407; 95% CI, −0.459, −0.355) and former smokers (β = −.093; 95% CI, −0.121, −0.064) had significantly lower IC scores compared to never smokers (Table 2). A similar pattern was observed across smoking frequency: individuals who smoked occasionally had lower IC than never smokers (β = −.123; 95% CI, −0.230, −0.017), and daily smokers had even lower IC scores than never smokers (β = −.403; 95% CI, −0.463, −0.343). Regarding the effect of the number of cigarettes smoked per day, a significant inverse association with IC was observed. Specifically, those who smoked 26+ cigarettes per day had a statistically significantly lower IC score compared to those who smoked 1-5 cigarettes a day (β = −.770; 95% CI, −1.106, −0.435). Likewise, passive smoking at home was inversely associated with IC: participants exposed once a week or less had lower IC than those never exposed (β = −.283; 95% CI, −0.373, −0.193), and those exposed daily or almost daily had even lower IC (β = −.422; 95% CI, −0.512, −0.332). Additional details are provided in Table 2 and Figure S1.

#### Sleep duration and quality

Several sleep-related variables had significant associations with IC. In general, longer average daily sleep duration (assessed as a continuous variable in hours per day) was positively associated with IC (β = .043; 95% CI, 0.031-0.054). Compared to individuals who reported sleeping within the recommended 7-9 h per night, those sleeping less than 7 h had significantly lower IC scores (β = −.133; 95% CI, −0.161, −0.105), while those sleeping more than 9 h had even lower IC scores (β = −.258; 95% CI, −0.392, −0.124), as shown in Figure S2. Individuals who had a higher frequency of daytime drowsiness had, on average, lower IC scores compared to those who never experienced such difficulty (details in Table 2). Satisfaction with current sleep patterns was also positively associated with IC: individuals who reported being very satisfied with their sleep had, on average, 0.12 higher IC scores than those who were very dissatisfied (β = .012; 95% CI, 0.042-0.198).

#### Physical activity

**Figure 2:**
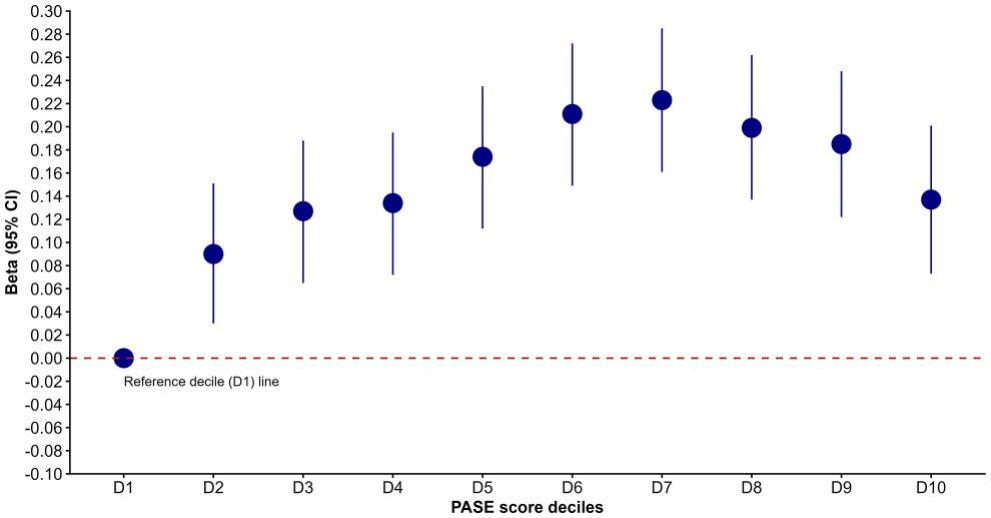
Association between physical activity (measured by PASE total score, grouped in deciles) and IC in the CLSA cohort. The *x*-axis represents deciles (D; D1 [lowest] to D10 [highest]) of the PASE total score, and the *y*-axis indicates the beta coefficients for the association of IC with PASE total score for individuals with high physical activity (PASE score deciles D2-D10) compared to those with the lowest physical activity levels (D1, PASE score decile 1 as a reference category — indicated by the horizontal dashed line or zero line). CLSA, Canadian Longitudinal Study on Aging; IC, intrinsic capacity; PASE, Physical Activity Scale for the Elderly.

Physical activity, measured using the PASE score, was positively associated with IC. Higher total PASE scores were significantly associated with higher IC scores (β = .0006; 95% CI, 0.0004-0.0008). The decile-based analysis of PASE scores demonstrated a clear dose–response relationship, with individuals with higher PASE score (in the second to tenth deciles) having significantly higher IC scores compared to those with the lowest physical activity score (in the first decile) (Figure 2). The association was strongest in the middle to upper deciles, suggesting that even moderate levels of physical activity may contribute significantly to higher IC.

#### Polygenic scores

**Figure 3:**
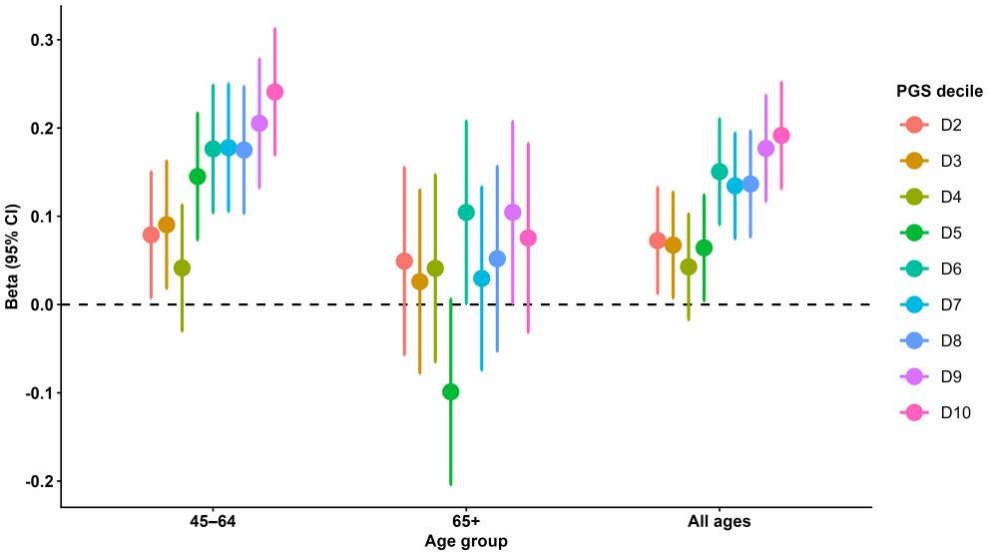
Association of PGS for IC with the general IC score, stratified by age group. Shown in the figure are linear regression coefficients (beta [β] represented by each point, with 95% CIs represented by whiskers) for individuals with a higher polygenic load (deciles D2-D10) compared with those with the lowest polygenic load (D1, dashed horizontal line at β = 0), plotted separately for the whole sample and age-stratified samples. IC, intrinsic capacity; PGS, polygenic scores.

The PGS-IC was associated with higher IC, with progressively greater β coefficients observed across higher deciles (D2-D9) relative to the reference decile, D1 (Figure 3). In stratified analyses by age group, we found that the associations were most pronounced in the 45-64 year-old age group but weaker and not statistically significant in the ≥65 year-old age group, with CIs for all deciles reaching or crossing zero (Figure 3).

#### Socioeconomic or lifestyle factors and PGS interaction

Our analysis revealed statistically significant interaction effects on IC of the socioeconomic or lifestyle factors and PGS-IC. Specifically, the effects of adherence to the Mediterranean diet (β = −.003; 95% CI, −0.006, −0.0002), education — high school completion (β = −.109; 95% CI, −0.211, −0.007), and long sleep duration in adults aged 45-64 years (β = .198; 95% CI, 0.023-0.373), as well as short sleep duration in individuals aged 65 years and older (β = −.095; 95% CI, −0.153, −0.036), were modulated by polygenic loading for IC. These results are provided in Table S4. When the effects of these factors on IC were compared across individuals in the low, middle, and high deciles of PGS-IC, the CIs for the effect estimates were overlapping across the PGS-IC categories. Details of the effect estimates of these factors on IC across the PGS-IC categories are presented in Table S5 and Figures S3-S5.

## Discussion

This study, for the first time, provided a comprehensive examination of how socioeconomic, lifestyle, and genetic factors jointly shape IC, revealing several important insights. Significant associations were identified between IC and modifiable factors, including physical activity (PASE score), dietary quality (PURE and Mediterranean diet scores), smoking, sleep health, and key socioeconomic indicators. While our earlier study demonstrated an overall association between PGS-IC and IC,^8^ the current analysis shows that the effect is evident in younger adults, whereas in older adults, it is not statistically significant. Importantly, significant interaction effects with PGS were observed for Mediterranean diet adherence (whole sample), educational attainment (45-64 years), and sleep duration (long sleep in 45-64 years; short sleep in ≥65 years), suggesting that the influence of the socioeconomic and lifestyle factors may vary with genetic predisposition to IC. Together, these findings extend previous work by clarifying both the independent and interaction effects of genetic and lifestyle factors in shaping IC.

Age-dependent pattern of PGS-IC associations with IC, whereby associations are evident in younger adults but not in older ones, may suggest that genetic predisposition plays a stronger role earlier in life, whereas in older age, prolonged exposure to lifestyle factors and related conditions exerts greater influence. This aligns with prior evidence on related traits such as functional ability and cognitive traits, where genetic effects have been shown to decline with age.^38^^,^^39^ Such a reduction may mainly reflect age-related alterations in gene expression and genetic effects across the lifespan.^39^^,^^40^

The interaction between adherence to a Mediterranean-type diet and PGS-IC indicated greater dietary benefit for individuals with lower genetic loading for IC. This finding points to the potential value of diet as a modifiable factor to help preserve IC, particularly in those with low genetic loading for IC. Comparable evidence has recently been reported for hand grip strength, where leisure-time physical activity exerted a stronger positive effect among individuals with lower PGS for grip strength.^41^ In line with this, adherence to the Mediterranean diet has been shown to provide greater benefit for those at high genetic risk of macular degeneration.^42^ Similar differential effect of diet with greater benefit for those with high genetic risk had also been reported in the context of other traits. For example, fruit and vegetable intake was found to confer greater protection against obesity among individuals genetically predisposed to higher BMI and weight gain.^43^

Education, among younger adults (45-64 years), had a negative interaction effect on IC (ie, greater effect (benefit) of education for those who had low genetic loading). This is in line with recent evidence, which reported that higher educational attainment attenuates the risk of dementia among those with high genetic predisposition for dementia.^44^ Comparable to our finding on sleep duration, a higher benefit of sleep quality (sleep consolidation) on cognitive measures was reported among individuals at higher genetic risk for Alzheimer’s disease.^45^

Despite the significant interaction effects detected for diet, sleep, and education, comparisons through separate regression analyses across the low, middle, and high PGS groups revealed only suggestive patterns, as CIs overlapped and statistical significance was not achieved. This may be due to reduced sample size in the subgroup analysis, which may have led to reduced statistical power to detect small effect sizes. For other socioeconomic and lifestyle factors, no evidence of interaction effects on IC was observed between the socioeconomic or lifestyle factors and PGS. These non-significant results may be partly attributable to limited statistical power, as robust detection of PGS × environment interactions typically requires very large sample sizes, a challenge well-documented in recent methodological works^46–49^ and observed in studies of cognition^50^ and cardiometabolic traits, even across large cohorts.^51^ To better estimate both the main and interaction effects of PGS, much larger samples will be required to detect effects with precision and to determine whether they hold sufficient strength to inform future clinical translation. Replication in independent cohorts will also be essential to confirm these preliminary findings. Accordingly, the present results should be interpreted as an initial step toward understanding the interplay between genetic susceptibility and modifiable factors influencing IC.

Our association tests with socioeconomic and lifestyle factors reaffirmed the beneficial effects of a healthier lifestyle and higher SES for IC. Among these, physical activity showed a strong positive association with IC, consistent with previous studies,^52^^,^^53^ and this is the first study to examine this relationship using the PASE scale, a validated tool for older adults. Our findings align with those from large cohort studies such as MAPT and SAGE, which link physical activity to higher baseline IC and slower decline,^54^^,^^55^ and with intervention trials showing that exercise improves mobility and mental well-being, which are key IC domains.^56^ The robust association may reflect the broad physiological benefits of physical activity in promoting healthy longevity by acting on biological aging processes.^57^

Dietary quality was also strongly associated with IC, with a strong dose-dependent pattern showing incremental benefits with increasing PURE and Mediterranean diet scores. These findings add to a growing body of literature highlighting the role of a quality diet in preserving functional ability and healthy longevity.^13^^,^^58^^,^^59^ Unlike earlier studies that focused on specific dietary components or pattern types,^60^^,^^61^ our use of composite indices such as PURE and Mediterranean dietary scores provides a more standardized assessment of dietary quality and its broader impact on IC. The beneficial effects on IC likely act through multiple pathways, including improved metabolic and cardiovascular health,^16^ reduced systemic inflammation,^62^ and preservation of cognitive function^63^^,^^64^ and biological aging,^15^ consistent with multi-omics evidence for the benefits of calorie-restriction in promoting healthy biological aging.^65^ Several clinical trials have also demonstrated that Mediterranean-style diets improve resilience and cognitive functioning^66^^,^^67^ and reduce frailty in older populations.^58^^,^^68^^,^^69^

Sleep quality and duration were associated with IC: optimal duration (7-9 h), greater sleep satisfaction, and less daytime sleepiness related to higher IC, consistent with prior works.^70–73^ We also reaffirmed inverse associations of sleep duration at both extremes (>10 h and <6 h) with IC.^72^

Our findings about smoking cigarettes showed its detrimental effects on IC; former smokers had lower IC than never-smokers, with current smokers having an even lower IC, indicating the benefits of both smoking cessation and avoidance of smoke for preserving IC. These findings align with evidence on the detrimental impact of tobacco on physical and cognitive health.^53^^,^^74–77^

Unsurprisingly, socioeconomic status also contributed to variability in IC: higher education and income were associated with higher IC, potentially through their contribution to building cognitive reserve, healthier behaviors, and better access to resources.^1^^,^^53^^,^^78–82^ Social engagement, especially cognitively and emotionally stimulating activities such as playing games or music, was likewise related to higher IC, consistent with prior studies demonstrating their impact in protecting against functional decline by enhancing cognitive and emotional resilience.^83–85^ Our findings were also in line with the WHO report that health outcomes consistently follow a social gradient, with lower socioeconomic position associated with poorer health across all levels of income.^86^

The main strength of this study lies in the novel findings of gene–environment interplay, by computing comprehensive composite measures of diet and a physical activity scale, specifically validated for the older population with IC. However, there are a few limitations to consider while interpreting the findings. First, because the CLSA sample is predominantly composed of individuals with European genetic ancestry, any attempt to generalize these findings to other populations requires replication in more diverse cohorts. Second, the gene–environment analyses generally demand a large sample size, and we anticipate that we could not detect the interaction effects on IC of some of the lifestyle and socioeconomic factors with PGS-IC due to sample size constraints. Third, the results reported in this study are based on cross-sectional associations, and longitudinal analyses would be required to establish direct cause-and-effect relationships between IC and the studied socioeconomic and lifestyle factors. Despite these limitations, our work adds to the literature by documenting associations of composite diet and physical-activity measures with IC and by showing that socioeconomic and lifestyle factors exert interaction effects with PGS-IC.

Taken together, our findings indicate that genetic predisposition to IC, particularly in midlife (45-64 years), is associated with IC and may buffer or attenuate the effects of socioeconomic and lifestyle factors. Specifically, novel significant interaction effects on IC were detected with education, diet, and sleep health. We also identified strong associations of IC with comprehensive composite measures of diet and physical activity and reaffirmed established links with key socioeconomic factors (education, income, employment, and social engagement) and lifestyle behaviors, notably sleep health and smoking. These results may inform future research aimed at understanding how sociodemographic and lifestyle factors may improve IC across different genetic profiles, contributing to better health promotion or preventative strategies across the lifespan to increase quality of life years. Future studies using larger and more diverse samples, harmonized IC measures, longitudinal designs, and integrative multi-omics approaches will be essential to clarify temporal relationships and underlying biological pathways.

## Supplementary Material

glag057_Supplementary_Data

## Data Availability

Data are available from the Canadian Longitudinal Study on Aging (www.clsa-elcv.ca) and the UK Biobank for researchers who meet the criteria for access to de-identified data.
