# Supplementary material for: Associations and interaction effects of socioeconomic, lifestyle, and genetic factors on intrinsic capacity
Source: J Gerontol A Biol Sci Med Sci. 2026 Feb 23;81(4):glag057. doi: 10.1093/gerona/glag057 (PMC13035082; doi:10.1093/gerona/glag057)
Supplement: glag057_Supplementary_Data [file glag057_supplementary_data.pdf]

Supplementary Material

Contents

Supplementary Methods 2

A. Prospective Urban Rural Epidemiological (PURE) study healthy diet score 2

B. Mediterranean diet score 3

C. Physical activity Scale for the elderly (PASE) 4

D. Intrinsic capacity (IC) score in CLSA 6

Table S1: Sociodemographic, economic and lifestyle characteristics of the study sample. 12

Table S2: Distribution of composite physical activity (PASE) and dietary (PURE and Mediterranean diet) scores. 14

Table S3: Association of individual and composite dietary intake with IC 14

Table S4: Socioeconomic and lifestyle factors that showed significant IC associations with PGSxE interaction 16

Table S5: Association of IC with socioeconomic and lifestyle factors stratified by PGS categories (for factors with significant interaction effect) 16

Figure S1: Distribution of IC scores across smoking status categories. 17

Figure S2: Distribution of IC scores by sleep category. 18

Figure S3: Association of IC with Mediterranean diet score across PGS groups (all ages). 19

Figure S4: Association of IC with high-school graduation by PGS and age groups. 20

Figure S5: Association of IC with sleep category across PGS groups, by age. 21

Supplementary Methods

A. Prospective Urban Rural Epidemiological (PURE) study healthy diet score

In this study, we restricted the prospective urban-rural epidemiological (PURE) healthy diet score based on the original PURE diet score (1) and the customized application of the method in the Canadian Longitudinal Study on Aging (CLSA)(2). As per this validated tool, we included seven healthy diet categories in calculating the score. We compute quantiles (0-4) of the daily frequency of intake, with quintile 0 representing the lowest intake and quintile 4 denoting the highest intake of the corresponding food. The total PURE health diet score is then the sum of the quintiles of daily intake frequency for the seven food groups, yielding a score of 0 (worst diet) to 28 (best diet). For food groups where more than one food is included, we add the daily frequency of intake for all included and then compute the quintiles, which then represent the overall intake frequency of the corresponding food group. The details are in the table below.

Table 1: Food groups and food items included for computing the PURE healthy diet score.

Food group	Included foods	Code in CLSA
**Fruits**	Fruits	NUT_FRUT_NB_COM
**Vegetables**	Green salads, Carrots, and Vegetables	NUT_GREEN_NB_COM<br>NUT_CRRT_NB_COM<br>NUT_VGOT_NB_COM
**Legumes**	Legumes	NUT_LEGM_NB_COM
**Nuts**	Nuts	NUT_NUTS_NB_COM
**Fish**	Fish	NUT_FISH_NB_COM
**Dairy**	Cheese (regular and low-fat)<br>Yoghurt (regular and low-fat)<br>Milk (whole milk, skim milk, and calcium fortified milk intake)	NUT_LWCS_NB_COM<br>NUT_CHSE_NB_COM<br>NUT_LWYG_NB_COM<br>NUT_YOGR_NB_COM<br>NUT_WHML_NB_COM<br>NUT_LFML_NB_COM<br>NUT_CAML_COM
**Red Meats, pork, and chicken**	Beef, pork, veal, lamb, game, and chicken intake	NUT_MEAT_NB_COM<br>NUT_MTOT_NB_COM<br>NUT_CHCK_NB_COM

Abbreviations: *PURE = Prospective urban rural Epidemiological study. CLSA=Canadian Longitudinal Study on Ageing.*

B. Mediterranean diet score

The Mediterranean diet score was calculated using dietary intake data from the Short Diet Questionnaire (SDQ) administered in the Canadian Longitudinal Study on Aging (CLSA), based on previously established methods (3, 4). Reported frequencies of food consumption were converted into estimated times per day. The score was constructed using ten food and beverage groups categorised as either beneficial or detrimental in alignment with the Mediterranean dietary pattern (4).

Beneficial components included whole grains, fruits, vegetables, legumes and nuts, potatoes, and fish. These were scored positively, with higher consumption associated with higher scores. Detrimental components—meat and meat products, poultry, full-fat dairy products, and alcohol—were scored inversely, so that higher consumption resulted in lower scores. Specifically, for alcohol, lower consumption was coded to have a higher score, then no consumption follows, and then the incrementally higher consumption assumes the least values.

Each component contributed 0 to 5 points (which were quintiles of daily frequency of intake), resulting in a total Mediterranean diet score ranging from 0 to 50, with higher scores indicating greater adherence to the Mediterranean dietary pattern. Details of the variables used in the calculation are provided in the following table.

Table 2: Food groups and included food items used to construct the Mediterranean diet score, classified as beneficial (+) or detrimental (–).

Food group	Food items included	Beneficial (+) or detrimental (-)	Codes in CLSA
**Whole grains**	High fibre breakfast cereals, whole wheat breads, bran breads, multigrain breads, and ryebreads	+	NUT_FBR_NB_COM<br>NUT_BRD_NB_COM
**Fruits**	Fruits	+	NUT_FRUT_NB_COM
**Vegetables**	Green Salads, carrots, and all other vegetables except potatoes	+	NUT_GREEN_NB_COM<br>NUT_CRRT_NB_COM<br>NUT_VGOT_NB_COM
**Legumes and Nuts**	Legumes, Nuts	+	NUT_LEGM_NB_COM<br>NUT_NUTS_NB_COM
**Potatoes**	Potatoes	+	NUT_PTTO_NB_COM
**Fish**	Fish	+	NUT_FISH_NB_COM
**Full-fat dairy**	Cheese, Yoghurt, and whole milk	-	NUT_CHSE_NB_COM<br>NUT_YOGR_NB_COM<br>NUT_WHML_NB_COM
**Meats**	Beef, Pork, and other meats (veal, lamb, game)	-	NUT_MEAT_NB_COM<br>NUT_MTOT_NB_COM
**Poultry**	Chicken	-	NUT_CHCK_NB_COM
**Alcohol**	Alcohol	-	ALC_FREQ_COM

C. Physical activity Scale for the elderly (PASE)

The Physical Activity Scale for the Elderly (PASE) was used to assess participants' physical activity over the past seven days of the assessment, incorporating leisure, household, and occupational domains. The score was computed from 12 self-reported items using the original PASE scoring manual (5, 6)), with adaptations for the CLSA data structure (7). For five activities (walking, light sport, moderate sport, strenuous sport, and strength exercise), scores were calculated using frequency × duration × activity-specific weight. Frequency and duration responses were translated to numerical values as follows:

- **Frequency to days/week**
    - Never = 0
    - Seldom (1–2 days/week) = 1.5
    - Sometimes (3–4 days/week) = 3.5
    - Often (5–7 days/week) = 6
- **Duration in hours/day**
    - Less than 30 minutes = 0.25

- 30 minutes to 1 hour = 0.75
- 1–2 hours = 1.5
- 2–4 hours = 3
- 4 or more hours = 4

For work-related activity, reported hours per week (capped at 40 hours) were converted to hours per day and scored only if the work involved physical effort (categories 2-4 of the intensity of work question). The remaining six items-housework, home repairs, yard work, outdoor activity, and caregiving-were binary and scored using fixed weights. The total PASE score was the sum of all component scores, with higher values indicating greater physical activity. The list of variables and scoring details is provided in the table below.

Table 3: Components of PASE score calculation: activity types, included variables (participation and duration) and corresponding PASE weights.

Physical activity	Variables we used to calculate the score	PASE weight
**Walking**	Average hours per day spent walking (PA2_WALKHR_MCQ) and Frequency of taking a walk outside (PA2_WALK_MCQ).	20
**Light sports**	Average hours per day engaged in light sports (PA2_LSPRTHR_MCQ) and Frequency of participation in light sports (PA2_LSPRT_MCQ)	21
**Moderate sports**	Average hours per day engaged in moderate sports (PA2_MSPRTHR_MCQ) and Frequency of participation in moderate sports (PA2_MSPRT_MCQ)	23
**Strenuous sports**	Average hours per day engaged in strenuous sports (PA2_SSPRTHR_MCQ) and Frequency of participation in strenuous sports (PA2_SSPRT_MCQ)	23
**Strength exercise**	Average hours per day exercised to increase muscle strength and endurance (PA2_EXERHR_MCQ) and its frequency (PA2_EXER_MCQ)	30
**Work involving PA (voluntary or paid)**	Hours worked for pay or as a volunteer (PA2_WRKHRS_NB_MCQ) and the Amount of physical activity required on the job or volunteering (PA2_WRKPA_MCQ)	21
**Light housework**	Engaged in light housework (PA2_LTHSWK_MCQ)	25
**Heavy housework**	Engaged in heavy housework or chores (PA2_HVYHSWK_MCQ)	25
**Home repairs**	Engaged in home repairs (PA2_HMREPAIR_MCQ)	30
**Heavy outdoor**	Engaged in lawn work or yard care (PA2_HVYODA_MCQ)	36
**Light outdoor**	Engaged in outdoor gardening, sweeping the balcony or the stairs (PA2_LTODA_MCQ)	20
**Care giving**	Engaged in caring for another person (PA2_CRPRSN_MCQ)	35

Abbreviations: *PASE = Physical Activity Scale for the Elderly*

D. Intrinsic capacity (IC) score in CLSA

The IC score was derived in the CLSA in our previous study (8) using a similar approach to that we applied in the UK Biobank (9). We followed a stepwise approach. Initially, a list of 27 variables guided by our scoping review (10) and which were available in the CLSA data were selected and conceptually aligned with the variables used to develop and validate the IC score in our previous UK Biobank study (9).

Then, using exploratory factor analysis, indicators with factor loadings ≥0.2 were identified, retaining 14 variables for final analysis. In line with the methodology used in our previous UK Biobank study (9) and using these 14 variables, exploratory and confirmatory factor analyses were conducted using both conventional and bifactor approaches for comparison. The final IC score was computed using the bifactor factor analysis method as it yielded better goodness-of-fit statistics. Details of the variables used and a description of the IC scores are presented below in this supplementary material. Additional details on the exploratory and confirmatory factor analysis results, as well as the goodness of fit statistics when using conventional and bifactor models, are available in the supplementary material in our published paper (8).

Table 4: List of 27 variables initially selected to develop the IC score.

Cognitive capacity	Locomotive capacity	Psychological capacity	Sensory capacity	Vitality capacity
Immediate recall	Average hrs of walking per day	Anxiety disorder (yes/no)	Hearing rating (self-rating)	Haemoglobin concentration
Delayed recall	Frequency of moderate physical activity	CESD10 depression score	Hearing aid use (Yes/No)	Grip strength (average)
Reaction time	Standing Balance	Mood disorder (yes/No)	Hearing difficulty with background noise	Appetite
Mental alteration test (MAT score)	Gait speed (time-spent_4m-walk)	Average hours of sleep per day (last month)	Visual acuity (best eye)	Weight loss
Animal fluency test (AFT_score1)	Frequency of taking a walk out of home		Vision aid use	Forced expiratory volume in 1 second (FEV1)
Verbal fluency test (FAS total score)	Chair rise test			
	timed Up and Go test (TUG test)			

***Abbreviation**: CESD10 = Centre for Epidemiologic Studies Depression Scale-10 item, IC = intrinsic capacity.*

Table 5: List of the final 14 variables used to construct the IC score and description of their measurement.

Variable	Name in the CLSA	Measurement
**Immediate Recall**	COG_REYI_SCORE_COM	Measured using Rey Auditory Verbal Learning Test. Number of words (or variants) correctly recalled in 90 seconds - Immediate Recall.
**Delayed Recall**	COG_REYII_SCORE_COM	Measured using Rey Auditory Verbal Learning Test. Number of words (or variants) correctly recalled in 90 seconds - Delayed Recall.
**Verbal fluency (FAS-score)**	Sum of: FAS_F_SCORE_COM, FAS_A_SCORE_COM, FAS_S_SCORE_COM	This test measures verbal fluency using the Controlled Oral Word Association Test (COWAT), specifically summing responses for the letters F, A, and S. Each sum represents the total number of words associated with each letter within a designated time frame.
**Standing Balance**	BAL_BEST_COM	This is measured by the best attained time for the standing balance test in seconds (given 60 seconds maximum and stopped beyond this).
**Walking Pace**	WLK_TIME_COM	This is a timed 4-meter Walk Test measured in total time required to complete 4m (in seconds).
**Anxiety disorder**	CCC_ANXI_COM	Self-reported (Yes/No) question
**CESD-10 Score**	DEP_CESD10_COM	Centre for Epidemiological Studies Short Depression Scale (CES-D 10) score.
**Mood disorder**	CCC_MOOD_COM	Self-reported (yes/no)
**Hearing rating**	HRG_HRG_COM	Self-rated hearing (rated 1-5)
**Hearing aid use**	HRG_AID_COM	Self-reported use of any hearing aid
**Hearing difficulty with background noise**	HRG_NOIS_COM	Self-reported hearing difficulty in the presence of background noise
**Haemoglobin concentration**	BLD_Hgb_COM	Haemoglobin concentration in grams per decilitre
**Hand grip strength**	GS_EXAM_AVG_COM	Average hand grip strength of the left and right hands
**FEV1**	Maximum	Forced expiratory volume in 1 second, the maximum of all trials was obtained

Abbreviation*: CESD10 = Centre for Epidemiologic Studies Depression Scale-10 item.*

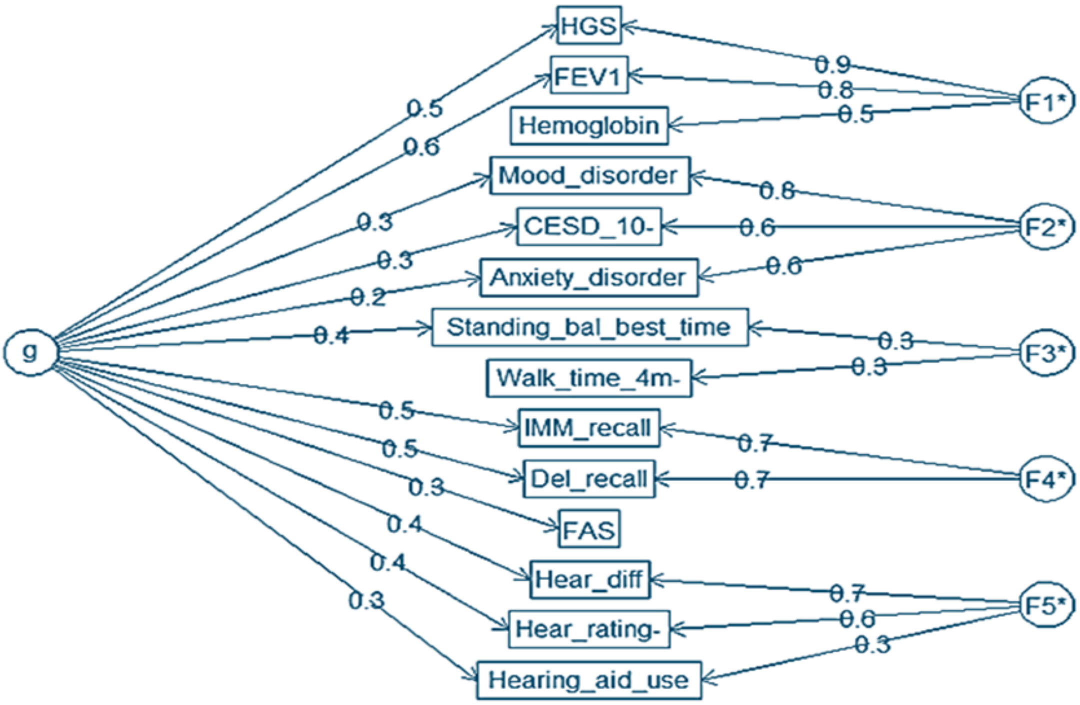

Figure 1. Bifactor CFA model. The g stands for general factor (IC), and F1 to F5 stand for the domains (specific factors) 1-5, respectively. Reproduced from Beyene et al., 2025 (11).

Abbreviations*: HGS = Hand grip strength, FEV1 = Forced expiratory volume in one second, CESD-10 = Centre for Epidemiologic Studies Depression Scale-10 item, IMM_recall = Immediate recall, Del_recall = Delayed recall, FAS = Verbal fluency using F-A-S letters.*

Distribution of IC score

After generating an intrinsic capacity score for the general and specific domains, we further explored their distributions, patterns of IC with age, and differences in IC score between the two sexes.

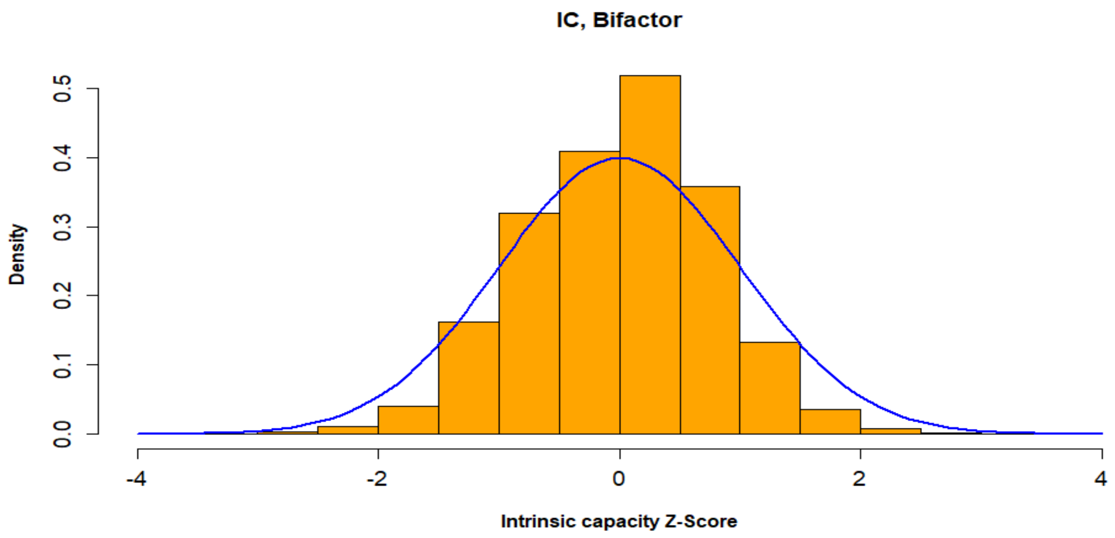

Figure 2: Histogram with Normal Curve for the IC General Score. Reproduced from Beyene et al., 2025 (11). ***Legend:*** *This histogram, overlaid with a normal curve, presents the distribution of the IC general score constructed from the bifactor confirmatory model. It has an approximately normal distribution.*

Abbreviations*: IC = intrinsic capacity*

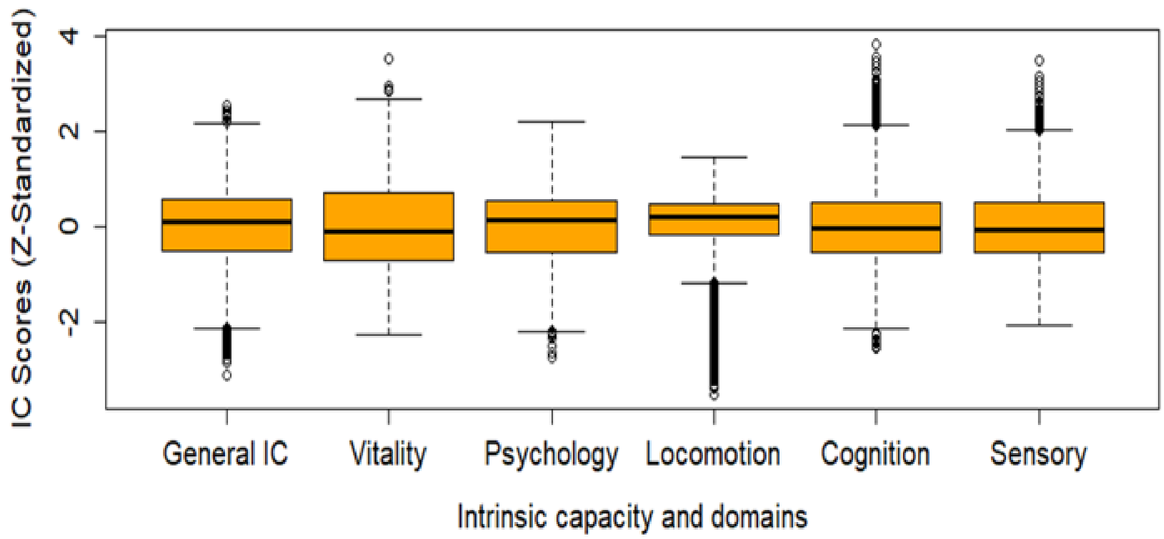

Figure 3: Distribution of the IC general score and domain-specific scores developed from the bifactor model. Reproduced from Beyene et al., 2025 (11).

Legend: *Box plots show the distribution of the intrinsic capacity general score and domain-specific scores derived from the bifactor confirmatory model. Boxes represent the interquartile range (25th–75th percentiles), the central line indicates the median, and whiskers indicate the range of values.*

Abbreviations*: IC = intrinsic capacity*

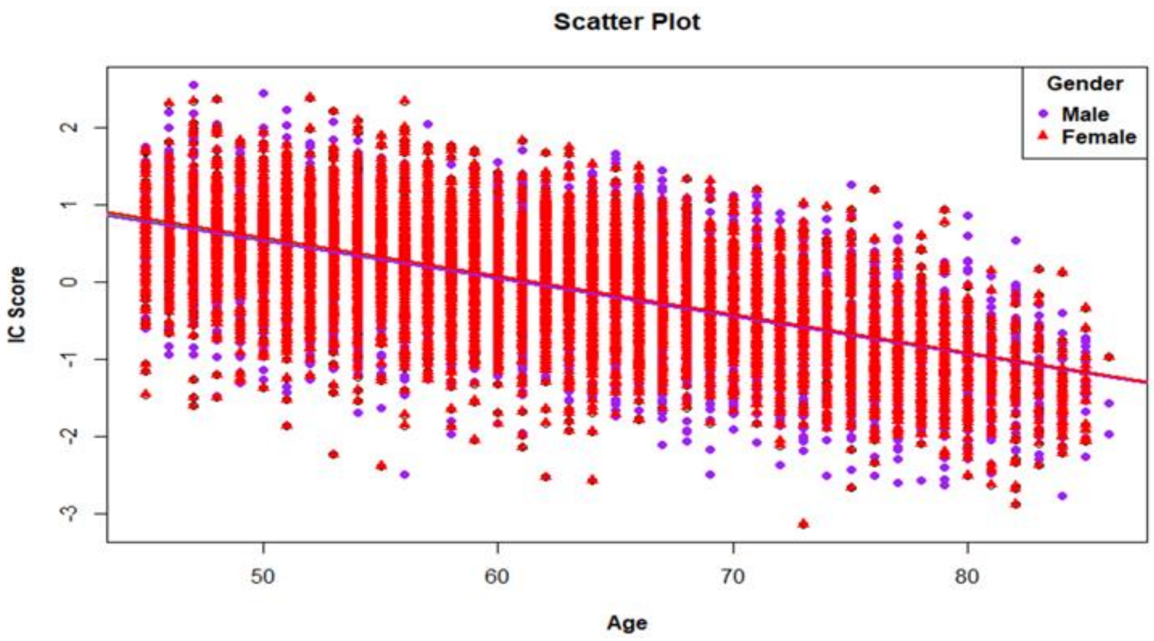

Figure 4: Intrinsic capacity score across age, by sex. Reproduced from Beyene et al., 2025 (11). ***Legend:*** *Scatter plot showing intrinsic capacity (IC) scores across age for males and females, with fitted linear regression lines. IC scores decline with increasing age in both sexes, with only minor differences between males and females across age groups.*

Abbreviations: *IC = intrinsic capacity*

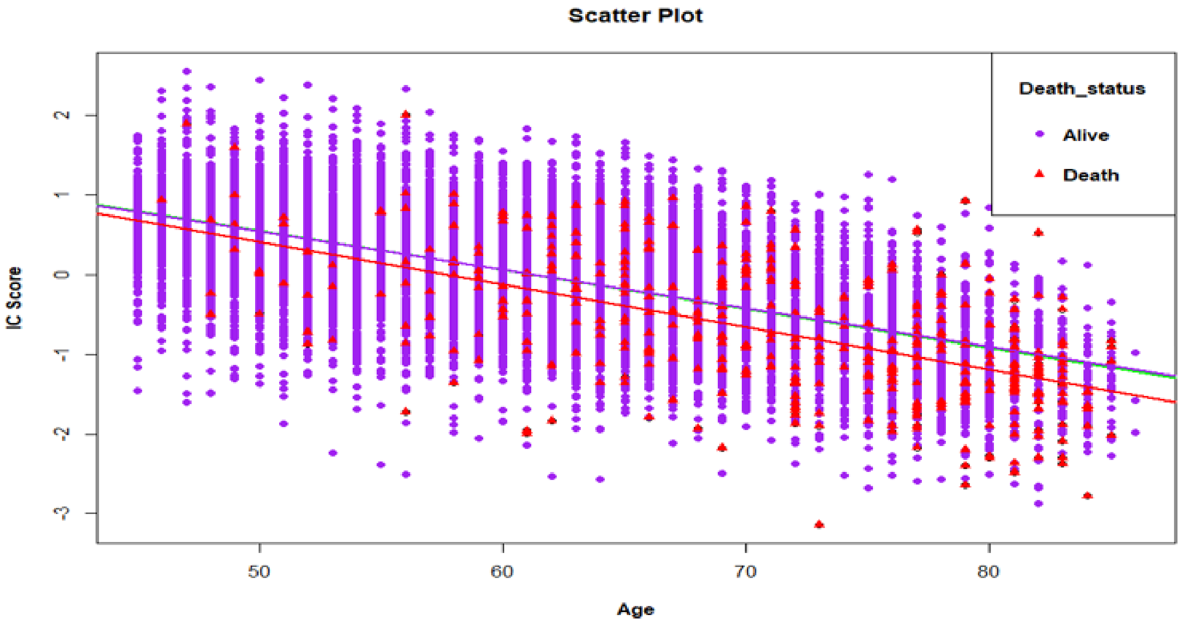

Figure 5: Intrinsic capacity score by age for deceased vs alive participants. Reproduced from Beyene et al., 2025 (11). ***Legend:*** *Scatter plot showing IC scores across age for deceased and alive participants, with fitted linear regression lines. Baseline IC scores are consistently lower among participants who died during follow-up compared with those who remained alive.*

Abbreviations: *IC = intrinsic capacity*

Table S1: Sociodemographic, economic and lifestyle characteristics of the study sample.

**Characteristics**	**Categories**	**N (%)**
**Age in years**	[45-53)	3283 (25.04)
	[53-60)	3322 (25.33)
	[60-68)	3461 (26.40)
	[68-86)	3046 (23.23)
**Sex**	Male	6453 (49.21)
	Female	6659 (50.79)
**Education**	< 2^0 school graduation	471 (3.40)
	2^0, no post-sec.	1092 (8.34)
	Some post-2^0 education	893 (6.82)
	Post-2^0 degree/diploma	10635(81.24)
**Ethnicity**	Caucasian	12333 (97.43)
	Asian	174 (1.38)
	African	79 (0.62)
	Hispanic	44 (0.35)
	Other ethnicity	28 (0.22)
**Country of birth**	Canada	10856 (82.80)
	Others	2255 (17.20)
**Personal income/year**	< $20,000	1552 (12.35)
	[$20,000, $50,000)	4330 (34.44)
	[$50,000, $100,000)	4607 (36.65)
	[$100,000, $150,000)	1294 (10.29)
	>$150,000	787 (6.26)
**Marital status**	Single	975 (7.55)
	Married/Living with a partner	9401 (72.82)
	Widowed	903 (6.99)
	Divorced	1278 (9.90)
	Separated	354 (2.74)

Table S1: Sociodemographic, economic and lifestyle characteristics of the study sample

Characteristics	Categories	N (%)
**Smoking status**	Former	5530 (42.17)
	Never	6579 (50.18)
	Current	1003 (7.65)
**Current frequency of cigarette smoking**	Never	7757
	Occasionally	217
	Daily	734
**Frequency of usual passive smoking exposure at home**	Never	9994 (94.32)
	Once a week or less	303 (2.86)
	Every day / almost every day	299 (2.82)
**Current (number of cigarettes smoked per day)**	1-5	116 (15.80)
	6-10	194 (26.43)
	11-15	178 (24.25)
	16-20	129 (17.58)
	21-25	84 (11.44)
	26+	33 (4.50)
**Sleep duration**	Recommended (7-9hrs)	8251 (62.93)
	Short (<7hrs)	4726 (36.04)
	Long (>9hrs)	135 (1.03)
**Frequency of difficulty staying awake during the day**	Never	8299 (63.36)
	Less than once a week	2605 (19.89)
	Once/twice a week	1230 (9.39)
	3-5 times a week	594 (4.54)
	6-7 times a week	370 (2.82)
**Satisfaction with current sleep pattern**	Very dissatisfied	468 (3.57)
	Dissatisfied	2688 (20.51)
	Neutral	2060 (15.72)
	Satisfied	5235 (39.96)
	Very satisfied	2658 (20.29)

Table S2: Distribution of composite physical activity (PASE) and dietary (PURE and Mediterranean diet) scores.

Variable	N	Observed range	Mean (SD)
**PASE total score**	13,112	1 - 488	149.11 (66.76)
**PURE Healthy diet score**	13,109	0 - 26	12.57 (4.71)
**Mediterranean diet score**	13,112	3 - 39	21.04 (4.87)

Abbreviations: *PASE = Physical Activity Scale for the elderly, PURE = Prospective Urban Rural Epidemiological study.*

Legend*: The lowest PASE score indicates the lowest physical activity level, while the highest score indicates the most strenuous physical activity level overall. Similarly, for PURE and Mediterranean diet scores, the lowest scores show the worst healthy diet and least adherence to Mediterranean type diet, respectively, whereas the highest score indicates the healthiest diet and highest adherence to Mediterranean type diet.*

Table S3: Association of individual and composite dietary intake with IC

Food/Diet type	Beta (95% CI)	P -Value
Pure fruit juice intake	-0.007 (-0.016, 0.001)	9.70E-02
Skim Milk intake	0.002 (-0.006,0.010)	6.63E-01
All other egg intake (except Omega-3)	-0.016 (-0.028, -0.004)	7.41E-03
Low-fat Cheese intake	0.023 (0.013, 0.033)	1.20E-05
Regular Cheese intake	0.011 (-0.002, 0.024)	9.36E-02
Butter or Regular Margarine intake	-0.019 (-0.029, -0.0099)	1.46E-04
Calcium fortified food intake	0.03 (0.011, 0.048)	2.07E-03
Calcium-fortified juice intake	-0.022 (-0.038, -0.007)	5.11E-03
Calcium fortified milk intake	-0.023 (-0.058, 0.011)	1.76E-01
Carrots intake	0.057 (0.036, 0.079)	1.77E-07
Chicken intake	-0.049 (-0.073, -0.025)	1.48E-04
Chocolate bar intake	0.008 (-0.002, 0.019)	1.29E-01
Fish Intake	0.059 (0.043, 0.075)	6.29E-13
French fries or pan-fried potatoes, poutine	-0.064 (-0.077, -0.053)	1.01E-24
Fruit intake	0.102 (0.078, 0.126)	2.84E-16
Green Salad intake	0.084 (0.065, 0.103)	2.35E-17
legumes: beans, peas, lentils intake	0.094 (0.080, 0.109)	2.79E-36
Milk-based dessert intake	-0.011 (-0.022, 0.001)	7.03E-02
Nuts, seeds, and peanut butter intake	0.079 (0.064, 0.095)	6.82E-24
Omega-3 Eggs intake	0.022 (0.012:0.033)	6.76E-05
other vegetables (except carrots, potatoes, or salad) intake	0.175 (0.152:0.198)	2.32E-50
Pastries intake	0.018 (0.006:0.032)	4.07E-03
Pates, cretons, terrines	-0.022 (-0.038:-0.007)	5.20E-03
Potatoes intake	-0.041 (-0.059: -0.023)	2.04E-05

regular vinaigrettes, dressings, and dips	0.019(0.006:0.032)	3.57E-03
Salty Snacks intake	-0.002 (-0.015:0.010)	7.11E-01
Sauces and gravies intake	-0.030 (-0.041: -0.019)	5.96E-07
Sausages, hot dogs, ham, smoked meat, and bacon	-0.042 (-0.055: -0.030)	1.61E-10
Whole Milk intake	-0.013 (-0.031:0.005)	1.45E-01
Low-fat yogurt intake	0.015 (0.006:0.023)	7.63E-04
Regular Yoghurt intake	0.016 (0.008:0.026)	3.90E-04
**Composite diet scores (continuous)**		
PURE healthy diet score	0.024 (0.021: 0.027)	4.93E-56
Mediterranean diet score	0.0186 (0.0157:0.0215)	4.90E-36
**Pure healthy diet score deciles (D1, ref)**		
D2: (6-8]	0.130 (0.069:0.190)	2.90E-05
D3: (8-10]	0.167 (0.111:0.223)	7.43E-09
D4: (10-11]	0.174 (0.108:0.239)	2.30E-07
D5: (11-13]	0.220 (0.165:0.274)	9.41E-15
D6: (13-14]	0.260 (0.195:0.325)	9.41E-15
D7: (14-15]	0.262 (0.196:0.328)	1.59E-14
D8: (15-17]	0.320 (0.260:0.377)	3.59E-26
D9: (17-19]	0.380 (0.312:0.441)	8.18E-30
D10: (19-28]	0.410 (0.344:0.476)	2.91E-33
**Mediterranean diet score deciles (D1, ref)**		
D2: (15-17]	0.129 (0.073:0.184)	6.91E-06
D3: (17-18]	0.14 (0.074:0.205)	2.98E-05
D4: (18-20]	0.152 (0.100:0.204)	1.43E-08
D5: (20-21]	0.15 (0.087:0.212)	4.33E-06
D6: (21-22]	0.198 (0.136:0.261)	7.68E-10
D7: (22-24]	0.243 (0.189:0.296)	2.51E-18
D8: (24-25]	0.245 (0.178:0312)	2.15E-12
D9: (25-27]	0.248 (0.186:0.310)	7.36E-15
D10: (27-50]	0.322 (0.263:0.381)	6.90E-26

Abbreviations: *CI = Confidence interval, D1-D10 = deciles 1 to 10.*

Legend: *All individual nutritional intake variables in the table are Likert scaled measurement of intake of the respective food types on five scales: 1. rarely/never, 2. Per Year, 3. Per Month, 4. Per week, 5. Per day, and were analysed in this paper as continuous variables. D2-D10 stand for the 2nd to 10th decile for the composite diet scores for which effect estimates and P-values were computed in comparison to the lowest diet score decile (D1).*

Table S4: Socioeconomic and lifestyle factors that showed significant IC associations with PGSxE interaction

		**All Ages**	**Age < 65**	**Age 65+**
**Variable/category**	**Categories**	**Beta (95% CI) – PGSxE**	**Beta (95% CI) – PGSxE**	**Beta (95% CI) – PGSxE**
**Graduated from high school**	Graduated vs not	-0.065 (-0.139,0.009)	**-0.109* (-0.211, -0.007)**	-0.021 (-0.153, 0.111)
**Mediterranean diet score**	Score (0-50)	**-0.003* (-0.006, -0.0002)**	-0.003 (-0.006, 0.001)	-0.002 (-0.007, 0.004)
**Sleep duration**	Short vs optimal	-0.021 (-0.049, 0.007)	0.012 (-0.024, 0.047)	**-0.095* (-0.153, -0.036)**
	Long vs optimal	0.026 (-0.110, 0.161)	**0.198* (0.023, 0.373)**	-0.191 (-0.459, 0.078)

Abbreviations: *IC = intrinsic capacity, PGS = Polygenic Scores, PGSxE = polygenic score with environment interaction, CI = confidence interval.*
Legend: *Values are interaction beta coefficients (β) with 95% confidence intervals (CI) from adjusted linear regression models. Separate regression models were fitted for the overall sample (All Ages) and stratified by age group (Age <65 and Age 65+). *Indicates statistically significant interaction effects after FDR correction (P_{adj} < 0.05).*

Table S5: Association of IC with socioeconomic and lifestyle factors stratified by PGS categories (for factors with significant interaction effect)

		**Low PGS**	**Middle PGS**	**High PGS**
**Variable/category**	**Categories**	**Beta (95% CI)**	**Beta (95% CI)**	**Beta (95% CI)**
**Graduated from high school**	Graduated vs not (Age: 45-64)	0.456 (0.131, 0.781)*	0.232 (0.117, 0.347)*	0.024 (-0.255, 0.302)
	Graduated vs not (Age: 65+)	0.348(-0.055, 0.752)	0.227(0.107, 0.348)*	0.408 (0.065, 0.751)*
**Mediterranean diet score**	Score (All Ages)	0.026(0.017, 0.035)*	0.018(0.015, 0.022)*	0.010 (0.002, 0.019)*
**Sleep duration**	Short vs optimal (Age:45-64)	-0.165 (-0.271, -0.060)*	-0.107 (-0.145, -0.071)*	-0.143 (-0.248, -0.039)*
	Short vs optimal (Age: 65+)	-0.088 (-0.252, 0.076)	-0.108 (-0.164, -0.052)*	-0.197 (-0.359, -0.034)*
	Long vs optimal (Age: 45-64)	-0.806 (-1.367, -0.244)*	-0.246 (-0.425, -0.067)*	0.134 (-0.360, 0.628)
	Long vs optimal (Age: 65+)	0.32 (-0.576, 0.641)	-0.301 (-0.553, -0.048)*	-0.770 (-1.877, 0.337)

Abbreviations: *IC = intrinsic capacity, PGS = Polygenic Scores, CI = confidence interval.*
Legend*: Values are beta coefficients (β) with 95% confidence intervals (CI) from adjusted linear regression models examining associations between socioeconomic and lifestyle factors and IC within strata of polygenic score (PGS) categories (Low, Middle, High). Low PGS corresponds to decile 1, Middle PGS to deciles 2–9, and High PGS to decile 10. * Indicates statistically significant associations after FDR correction (Padj < 0.05).*

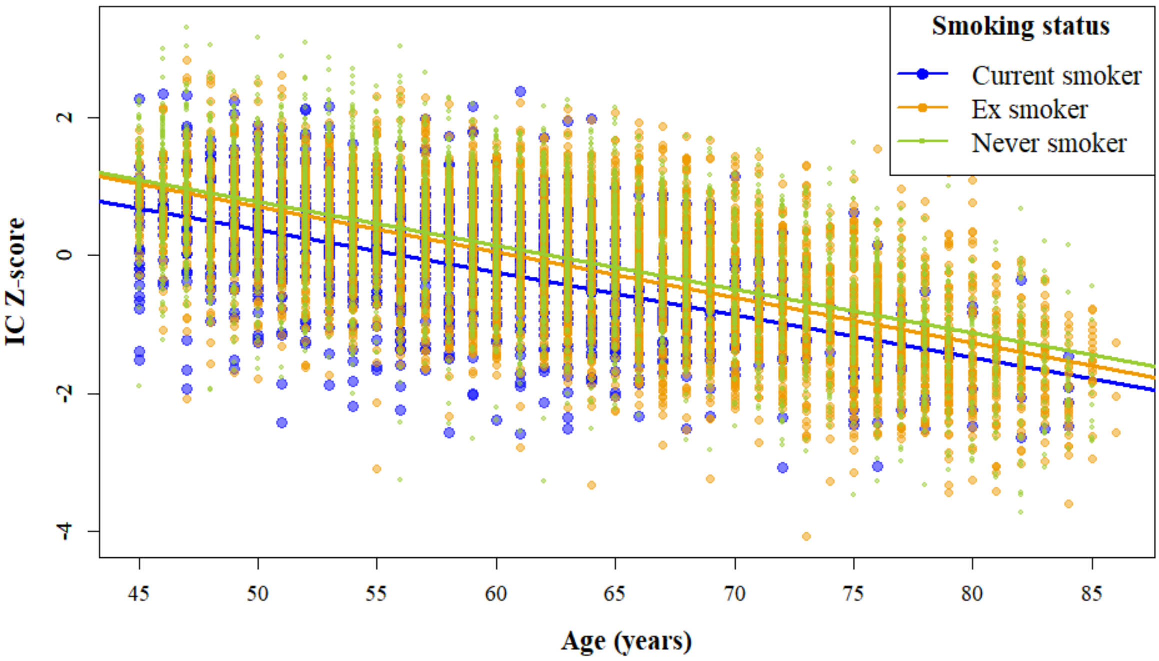

Figure S1: Distribution of IC scores across smoking status categories.

Legend: *The distribution of IC z-scores (y-axis) across age (x-axis), stratified by smoking status. Separate trend lines are presented for current smokers, ex-smokers, and never smokers. The figure illustrates a general decline in IC scores with increasing age, with lower scores observed among current smokers compared to ex- and never smokers across the age range.*

Abbreviations*: IC = Intrinsic capacity*

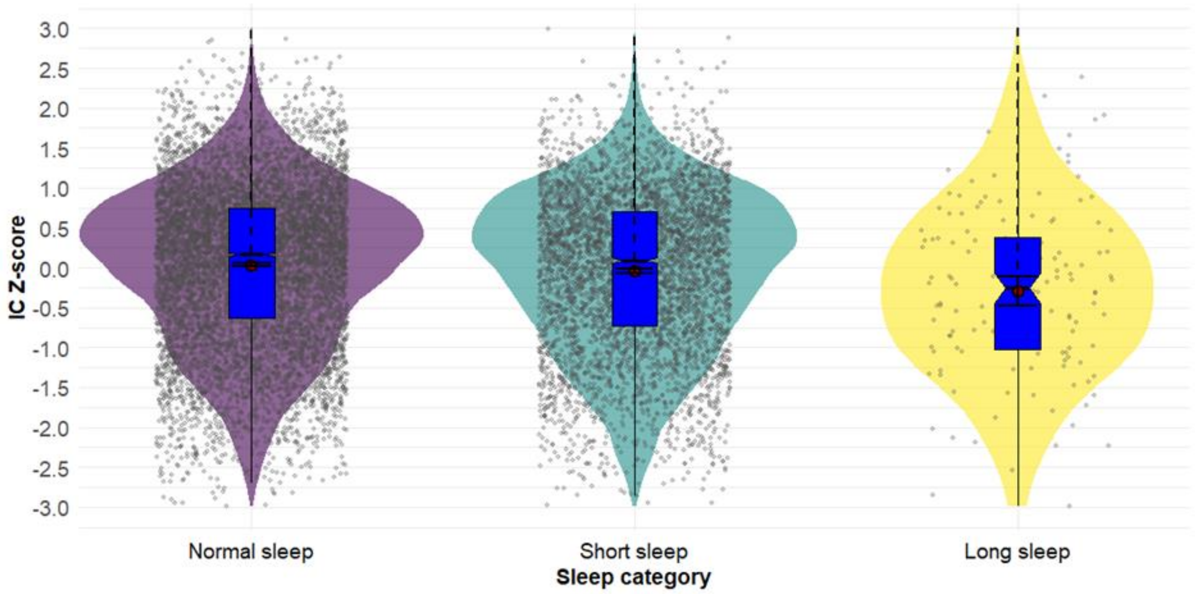

Figure S2: Distribution of IC scores by sleep category.

Legend: *This violin plot illustrates the distribution of IC Z-scores across three sleep duration categories: normal sleep (7–9 hours), short sleep (<7 hours), and long sleep (>9 hours). The y-axis represents the IC Z-score, while the x-axis indicates sleep categories. The width of each violin reflects the density of participants within a given IC score range. Overlaid box plots show the interquartile range and median IC scores for each group. The plot demonstrates lower median IC scores among individuals with short and long sleep durations compared to those with normal sleep duration.*

Abbreviations*: IC = Intrinsic Capacity*

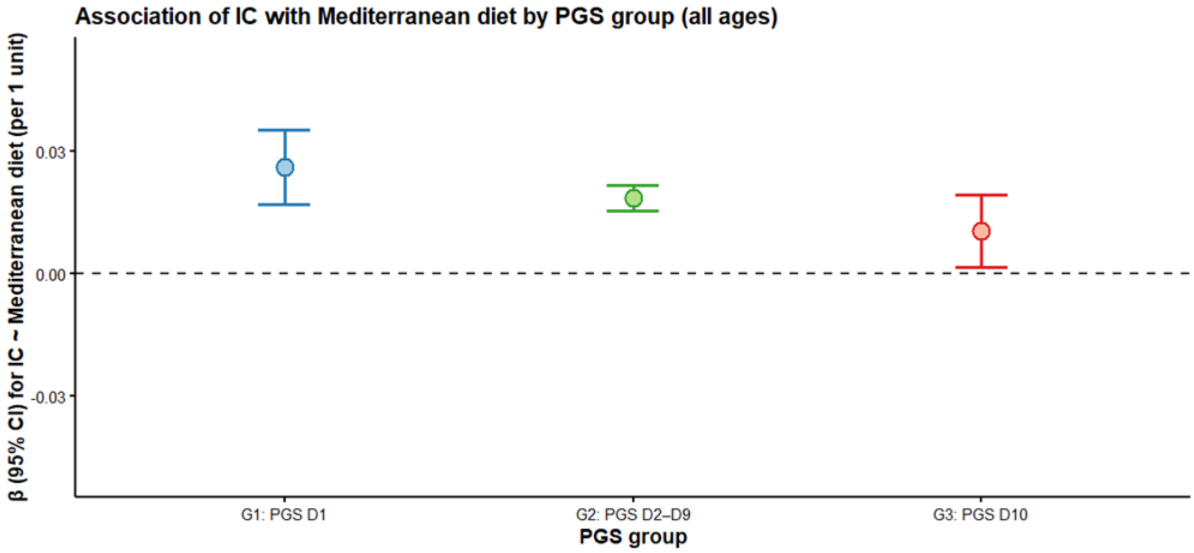

Figure S3: Association of IC with Mediterranean diet score across PGS groups (all ages).

Legend: *In the figure, points are linear regression coefficients (β) for IC per 1-unit increase in the Mediterranean diet score, estimated separately within three polygenic score (PGS) groups (G1: D1; G2: D2–D9; G3: D10). Vertical bars are 95% confidence intervals. The dashed horizontal line indicates β = 0.*

Abbreviations*: PGS = Polygenic scores, G1-G3 groups 1 to 3 using the polygenic score Deciles D1 -D10, IC = Intrinsic capacity.*

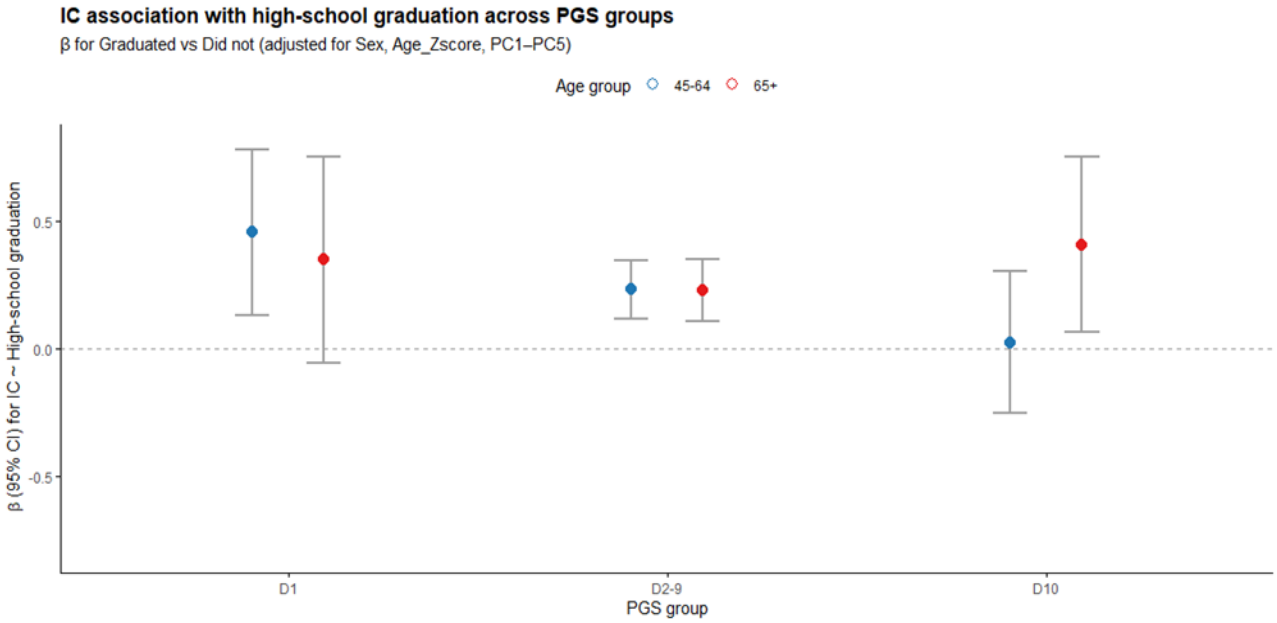

Figure S4: Association of IC with high-school graduation by PGS and age groups.

Legend: *Points show linear regression coefficients (β) for IC comparing Graduated vs Did not graduate high school, estimated separately within three polygenic score (PGS) groups (D1, D2–9, D10) and two age strata (45–64 in blue; 65+ in red). Vertical bars are 95% confidence intervals. The dashed horizontal line indicates β = 0.*

Abbreviations*: PGS = Polygenic scores, IC = intrinsic capacity, PC1-PC5 = principal components 1-5, CI = confidence interval, D1-D10 deciles 1 to 10.*

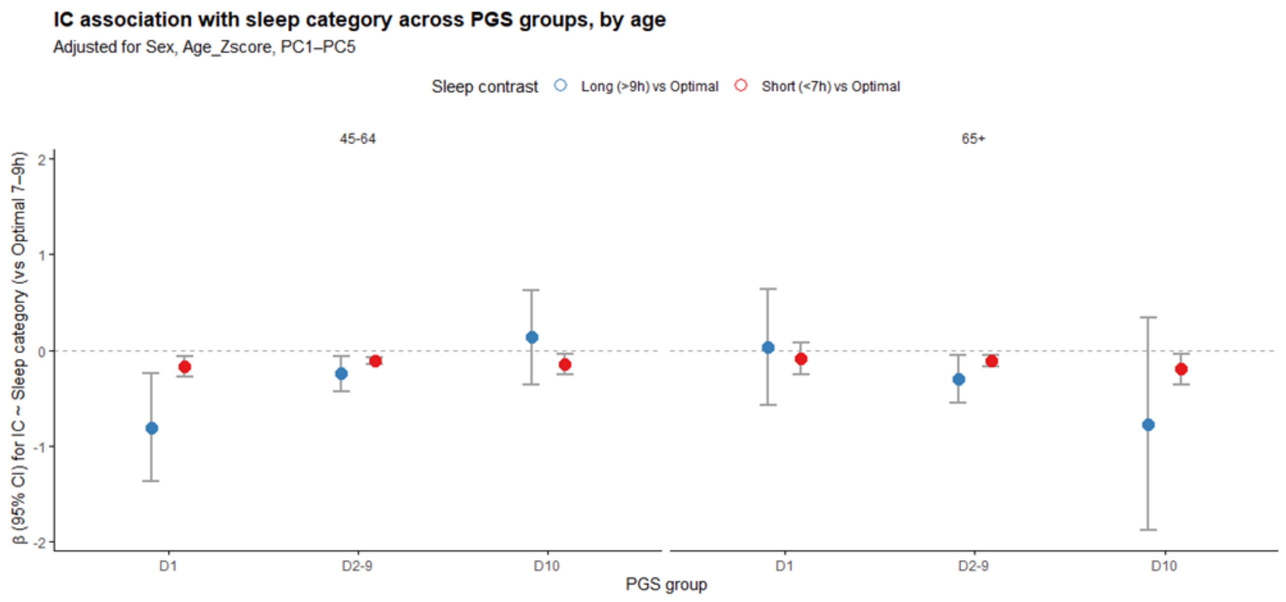

Figure S5: Association of IC with sleep category across PGS groups, by age.

Legend: *Points are linear regression coefficients (β) for IC comparing each sleep category with the optimal 7–9 h reference (Short <7 h vs Optimal and Long >9 h vs Optimal). Estimates are shown within three polygenic score (PGS) groups (D1, D2–9, D10) and for two age strata (45–64 and 65+; separate panels). Vertical bars indicate 95% confidence intervals, and the dashed horizontal line marks β = 0.*

Abbreviations: *PGS = Polygenic scores, IC = intrinsic capacity, PC1-PC5 = principal components 1-5, CI = confidence interval, D1-D10 deciles 1 to 10.*

References

1. Mente A, Dehghan M, Rangarajan S*, et al*. Diet, cardiovascular disease, and mortality in 80 countries. Eur Heart J. 2023;**44**:2560-2579. 10.1093/eurheartj/ehad269
2. Bassim C, Mayhew AJ, Ma J*, et al*. Oral health, diet, and frailty at baseline of the Canadian longitudinal study on aging. Journal of the American Geriatrics Society. 2020;**68**:959-966.
3. Vahid F, Wilk P, Bohn T. Longitudinal effects of diet quality on healthy aging - Focus on cardiometabolic health: findings from the Canadian longitudinal study on aging (CLSA). Aging Clinical and Experimental Research. 2025;**37**:157. 10.1007/s40520-025-03058-9
4. Aoun C, Papazian T, Helou K, El Osta N, Khabbaz LR. Comparison of five international indices of adherence to the Mediterranean diet among healthy adults: similarities and differences. Nutrition research and practice. 2019;**13**:333-343.
5. Washburn RA, Smith KW, Jette AM, Janney CA. The physical activity scale for the elderly (PASE): Development and evaluation. Journal of Clinical Epidemiology. 1993;**46**:153-162. https://doi.org/10.1016/0895-4356(93)90053-4
6. Institutes NER. PASE: Physical Activity Scale for the Elderly: Administration and Scoring Instruction Manual. New England Research Institutes Watertown, MA; 1991.
7. D'Amore C, Griffith L, Richardson J, Beauchamp M. Physical Activity Behaviour in Middle-Aged and Older Canadian Women and Men: An Analysis of the CLSA. medRxiv. 2025:2025.2003.2017.25323990. 10.1101/2025.03.17.25323990
8. Beyene MB, Visvanathan R, Alemu R*, et al*. A genome-wide association study identified 10 novel genomic loci associated with intrinsic capacity. medRxiv. 2025:2025.2002.2005.25321753. 10.1101/2025.02.05.25321753
9. Beyene MB, Visvanathan R, Ahmed M, Benyamin B, Beard JR, Amare AT. Development and validation of an intrinsic capacity score in the UK Biobank study. Maturitas. 2024;**185**:107976. https://doi.org/10.1016/j.maturitas.2024.107976
10. Beyene MB, Visvanathan R, Amare AT. Intrinsic Capacity and Its Biological Basis: A Scoping Review. The Journal of Frailty & Aging. 2024;**13**:193-202. https://doi.org/10.14283/jfa.2024.30
11. Beyene MB, Visvanathan R, Alemu R*, et al*. A genome-wide association study identified 10 novel genomic loci associated with intrinsic capacity. J Gerontol A Biol Sci Med Sci. 2025;**80**. 10.1093/gerona/glaf196
